# *Blepharostoma vietnamicum* (Marchantiophyta): A New Taxon from Indochina, the Unique Largest Species in the Genus

**DOI:** 10.3390/plants13223215

**Published:** 2024-11-15

**Authors:** Vadim A. Bakalin, Anna A. Vilnet, Van Sinh Nguyen, Seung Se Choi

**Affiliations:** 1 Laboratory of Cryptogamic Biota, Botanical Garden-Institute of the Far Eastern Branch of the Russian Academy of Sciences, Makovskogo Street 142, Vladivostok 690024, Russia; 2 Polar-Alpine Botanic Garden-Institute—Separate Subdivision of Federal Research Centre ‘Kola Science Centre’, Akademgorodok 18A, Apatity 184209, Russia; anya_v@list.ru; 3 Institute of Ecology and Biological Resources, Graduate University of Science and Technology, Vietnam Academy of Science and Technology, Hanoi 10072, Vietnam; vansinh.nguyen@iebr.vast.vn; 4 Team of National Ecosystem Survey, National Institute of Ecology, Keumgangro 1210, Seocheon 33657, Republic of Korea

**Keywords:** *Blepharostoma*, Trichocoleales, evolution, molecular genetics, Vietnam, Indochina

## Abstract

*Blepharostoma* is one of the most ancient extant liverwort genera, within which the genetic diversity is quite high, whereas the morphological diversity, owing to the supposed stasis, is quite low. Unusually large plants of this genus were collected in North Vietnam and are described here as new-to-science species via an integrative approach. The two studied specimens do not reveal variability in the sequenced ITS1-2 nrDNA and *trn*L-F cpDNA loci, are clearly separated from other species by the level of genetic distances, and maintain a stable position on the reconstructed phylogenetic trees. This species is characterized, in addition to the large overall size of the plants, by larger leaf segment cells and a mixed character of oil bodies (i.e., small homogeneous and larger finely papillose ones within one cell). A description of the new taxon; its diagnostic characteristics; photographs; and discussions regarding its ecology, morphological similarities, and potential distribution are provided.

## 1. Introduction

*Blepharostoma* is a predominantly Holarctic genus of liverworts that penetrates the Southern Hemisphere but does not reach the sub-Antarctic. The genus is the only member of the family Blepharostomataceae included in the order Trichocoleales [1]. This genus is very easy to recognize, even under a hand lens, because of its isophyllous structure and leaves consisting of 3–4 uniseriate segments that are united only very near the base. The latest survey of the diversity of the genus was performed by Bakalin et al. [2]. At present, *Blepharostoma* counts eight legitimately described species and two still undescribed taxa, one of which has a hybrid origin and undistinguished morphologically from *B. brevirete*, other—clearly distinct from nucleotide sequence data, but differentiated morphological features could not be described from the only specimen known for the species. Bakalin et al. [2] showed that within *Blepharostoma,* there is probably a phenomenon of stasis, in which initially (and long ago) divergent taxa were subsequently pressed into a number of similar ecological niches that resulted in morphological similarity; however, it is associated with high genetic dissimilarity. The same paper, which was based on a study of predominantly East Asian materials, revealed that the taxonomic diversity of the genus is significantly underestimated and that new taxa should be described. The characteristics that were confirmed to be usable in the morphological delimitation of species of the genus are the previously neglected features of the structure of oil bodies (which requires the study of living material—a labor-intensive and nearly impossible task, especially considering the ‘deposits’ of this genus in world herbaria), as well as the stem cross-section features and the relative length of the cells of leaf segments and the presence of protrusions from the transverse walls between the cells of the uniseriate segments. Paper [2] described five new species, and it is highly likely that further new taxa await discovery. All previously recognized and newly described taxa were studied, including molecular-genetic methods that make the comparison with further novelties somewhat easier than commonly in other groups.

While working in Vietnam, our attention was paid to the plants that were similar in appearance to *Blepharostoma* but were much larger, even resembling *Pseudolepicolea andoi* (R.M. Schust.) Inoue under the low magnification of a hand lens. A laboratory study of the plants immediately revealed that they were representatives of the genus *Blepharostoma*, but the species’ identity was not obvious. A discussion of this record and the systematic position of the observed plants is the main goal of this work.

## 2. Results

### 2.1. Molecular Phylogenetic Reconstruction

**Figure 1:**
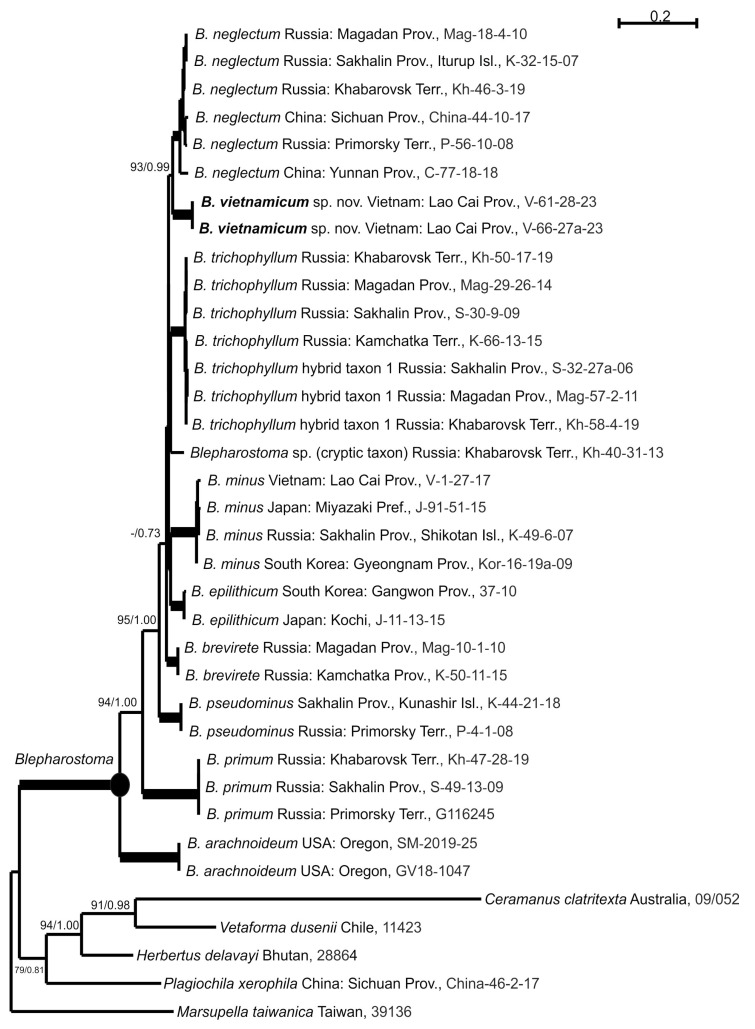
The phylogram for the genus *Blepharostoma* obtained via the maximum likelihood approach based on ITS1-2 nrDNA. Bootstrap support values and posterior probabilities greater than 50% (0.50) are shown. The phyla to the nodes with the 100/1.00 supports are in bold.

**Figure 2:**
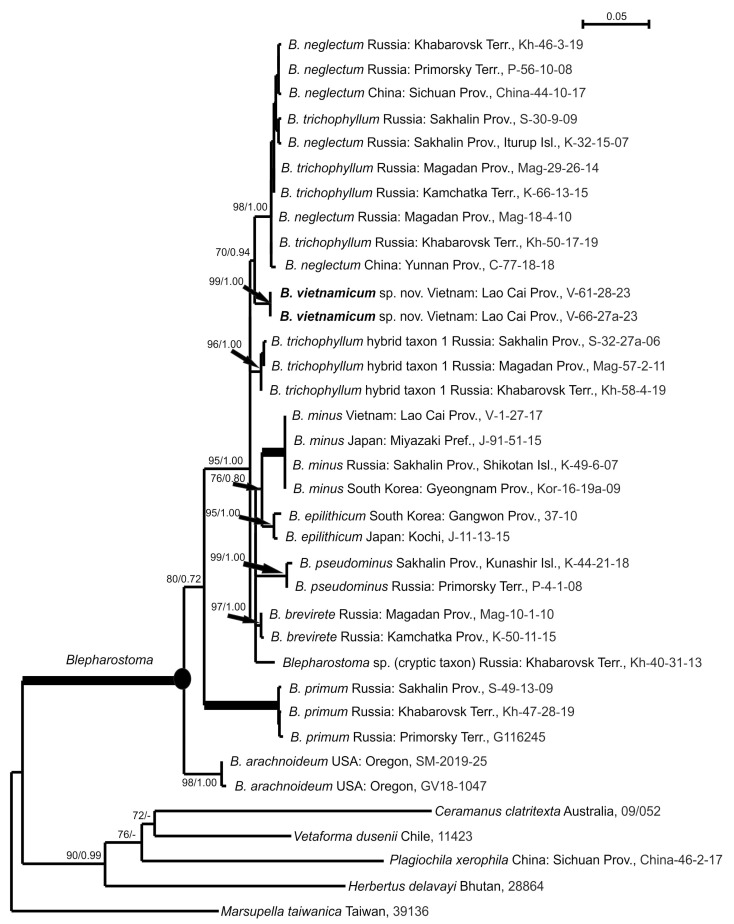
The phylogram for the genus *Blepharostoma* obtained via the maximum likelihood approach on the basis of *trn*L-F cpDNA. Bootstrap support values and posterior probabilities greater than 50% (0.50) are shown. The phyla to the nodes with the 100/1.00 supports are in bold.

For two tested *Blepharostoma* specimens from North Vietnam, the ITS1-2 and *trn*L-F sequence data were obtained and deposited into GenBank. The ITS1-2 and *trn*L-F datasets include 36 accessions belonging to the specimens mentioned in the Materials and Methods section and Table 1. The ITS1-2 dataset includes 945 positions, and *trn*L-F includes 588 positions. The arithmetic mean of log-likelihood achieved in the ML calculation of the ITS1-2 dataset is −6766.003; in both runs of the BA analysis, the values are −6799.52 and −6799.03. The topologies from both estimations of the ITS1-2 datasets are similar, and Figure 1 shows an ML tree with bootstrap support (BS) values from the ML analyses and Bayesian posterior probabilities (PP) from the BA. For the *trn*L-F dataset calculation, the arithmetic means of log-likelihoods are −2765.967 in the ML and −2798.31 and −2798.44 in the BA. Both analyses of the *trn*L-F dataset achieved identical topologies, and Figure 2 illustrates the ML tree with nodes supported by BA and PP.

The reconstructed tree topologies mainly agree with those published by Bakalin et al. [2]. The differences include 1) the unsupported sister relationship of *B. minus* and *B. epilithicum* that is opposite to the unresolved polytomy in the ITS1-2 topology; 2) the placement of the *B. trichophyllum* hybrid 1 clade separately from the clade of the sister related *B. minus* and *B. epilithicum* opposite their previous placement in one clade in the *trn*L-F topology; and 3) the placement of *B. pseudominus*, *B. brevirete* and the undescribed cryptic taxon in the unsupported polytomy opposite the subsequent divergence in the previous *trn*L-F estimation. Nevertheless, the clades corresponding to distinct species are separated by both DNA loci, but their relationships remain unclear. This could be the result of hybridization events or insufficient phylogenetic resolution of selected DNA markers, as well as the influence of new genetic units included in the current estimation. Both specimens from North Vietnam form a clade with the highest support from the estimations of the ITS1-2 (BS = 100%, PP = 1.00 or 100/1.00) and *trn*L-F (99/1.00) datasets (Figure 1 and Figure 2). This clade was a sister to the *B. neglectum* clade in terms of ITS1-2 (93/0.99) and *B. neglectum* + *B. trichophyllum* clade in *trn*L-F (70/0.94) analyses. Neither Vietnamese sample revealed variability in the ITS1-2 or *trn*L-F nucleotide sequences (Table 2). The level of their divergence from other *Blepharostoma* species is 6.8–20.1% in ITS1-2 and 2.4–8.7% in *trn*L-F, which is in accordance with the levels of genetic difference among species of the genus *Blepharostoma,* with the exception of taxa with ancient hybrid origins, namely *B. neglectum* and *B. trichophyllum* hybrid taxon 1, which have similarity in loci with parental inheritance from *B. trichophyllum* (Table 2). The stable distinct position on the phylogenetic trees and the level of nucleotide sequence divergence suggested that the identified genetic units could be associated with previously unknown taxa at the species level.

### 2.2. Taxonomic Treatment

Since both the morphological and molecular genetic comparisons unambiguously revealed a new taxon and considering the well-defined position of the specimens on phylogenetic trees, a morphological description of the plants was compiled on the basis of the studied specimens, and the holotype and paratypes of the new taxon were selected, which, in our opinion, deserve species ranking. A description of the new species is given below.

*Blepharostoma vietnamicum* Bakalin, Vilnet et S.S. Choi sp. nov.

**Figure 3:**
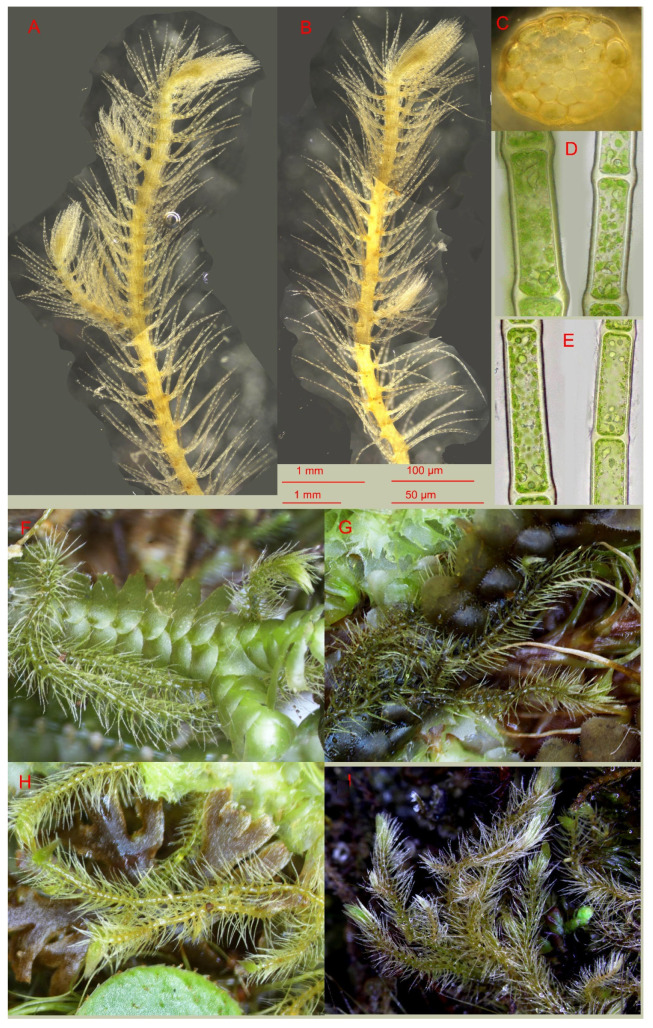
*Blepharostoma vietnamicum* Bakalin, Vilnet et S.S. Choi sp. nov.: (**A**,**B**) plant habit (photographed with dark field option); (**C**) stem cross section (photographed with dark field option); (**D**,**E**) leaf segment fragments; (**F**) plants in natural condition growing over *Bazzania himalayana* (Mitt.) Schiffn.; (**G**) plants in natural condition growing over *Scapania ornithopoides* (With.) Waddell; (**H**) plants in natural condition growing over *Riccardia* sp.; (**I**) plants in natural condition. Scales: upper 1 mm for (**A**,**B**); upper 100 µm for (**C**); lower 50 µm for (**D**,**E**); lower 1 mm for (**F**–**I**). (**A**–**D**) V-61-28-23; (**E**) V-66-27-23; (**F**) V-66-27a-23; (**G**,**H**) V-61-28-23; (**I**) V-17-2-18 (VBGI).

Plants large in comparison with other *Blepharostoma* taxa, 1.25–1.75 mm wide and 10–30 mm long, creeping over other liverworts, freely branched with branching of *Frullania*-type. Rhizoids not seen. Stem nearly straight (not flexuous), dorsal side cells 65–80 µm long and 28–35 µm wide, nearly rectangular, thin-walled, with small concave trigones, cuticle smooth; stem cross section transversely ellipsoidal, in well-developed shoots 120–130 µm high and 130–190 µm wide, external wall strongly thickened throughout, outer cells 17–25 × 20–30 µm elongate along cross section margin, with moderate in size, concave trigones, inward cells slightly smaller, 13–25 × 15–28 µm, thin-walled or walls slightly thickened, trigones small, concave. Leaves transversely inserted, consist of four unbranched segments, undivided zone one cell high, segments nearly straight, 900–1100 µm (12–15 cells) long; undivided zone composed by 4–6-gonal subisodiametric cells with thickened walls, 40–55 µm in diameter; cells of segments base 50–80 × 35–45 µm, becoming narrower and longer distally, in the segment middle 70–100 × 15–22 µm, slightly longer near apices, but two last cells of the segment commonly shorter, 30–45 × 5–10 µm; cuticle distinctly finely papillose in the segment middle; walls between segment cells projecting beyond the segment line and segment looks crenulate. Oil bodies in the middle cells of the leaf segments of two types: larger, finely papillose, 5–8 per cell, spherical 3–5 µm in diameter to oblong to 7 µm long; and small ‘oil drops’ less 1 µm in diameter, number is hard to calculate, presumable 15–40 per cell. Underleaves similar to leaves, but with segments somewhat shorter, in average 700–900 µm long, consisting of 10–14 cells, with average cell length in the segment middle 60–80 µm; cuticle finely papillose. Reproductive organs unknown. Figure 3.

Holotype: Northwest Vietnam, Lào Cai Province (22.29375° N 103.79854° E), 2842 m a.s.l. Sa Pa District, San Sả Hồ Commune, eastern spur of Phan Xi Pang Mt. Crooked *Rhododendron* forest with dense bamboo understory on a steep slope, open moist cliff. V.A. Bakalin, V-61-28-23 (16 May 2023), VBGI, isotype in KPABG.

Paratypes: North Vietnam, Lao Cai Province (22.30778° N 103.77361° E), 2846 m a.s.l. Sa Pa District, San Sa Ho Commune, Hoang Lien Range, Hoang Lien National Park, one of the ways to the Phan Xi Pan Peak. Thickets of *Sinobambusa* with many rocky outcrops and *Rhododendron* trees; partly shaded moist cliff crevice. V.A. Bakalin and K.G. Klimova, V-17-2-18 (03 April 2018), VBGI. The same (22.29538° N 103.80583° E), 2559 m a.s.l., eastern spur of Phan Xi Pang Mt., E-facing steep slope with mostly *Rhododendron* scattered forest and some *Abies fansipanensis* Q.P. Xiang, L.K. Fu and N. Li and bamboo understory, partly shaded, moist cliff. V.A. Bakalin, V-66-27a-23 (17 May 2023), VBGI, KPABG. The same (22.29444° N 103.80481° E), 2591 m a.s.l., the ridge line of the small spur with *Rhododendron* dominating forest and bamboo understory, partly shaded moist cliff. V.A. Bakalin, V-62-48a-23 (16 May 2023), VBGI.

Etymology: the species was named after the country where it was collected (Vietnam).

## 3. Discussion

### 3.1. Morphological Distinctions

*Blepharostoma vietnamicum* is much larger than any other species known within the genus. As reviewed by Bakalin et al. [2], most species of the genus range from 300 to 700 μm in width, and only the largest of them, *B. arachnoideum*, reaches 1 mm in width [3]. Thus, *B. vietnamicum* is nearly twice as large as the vast majority of other representatives. When comparing the difference in plant size, the lengths of the segments also differ; in *B. vietnamicum,* the segment lengths exceed 1 mm, whereas in other representatives of the genus, as a rule, they are no more than 400 μm long. The cell lengths in the middle of the segment usually do not exceed 60 µm in other representatives of the genus, whereas in *B. vietnamicum*, the length can reach 100 µm.

As shown earlier [2], one of the most important characteristics in the delimitation of species within the genus is the oil bodies (e.g., few, relatively large and botryoidal or homogeneous, numerous, and small). A unique feature of *Blepharostoma vietnamicum* is the combination of both large and small oil bodies within the same cell. Moreover, the large oil bodies do not have a botryoidal structure and are rather finely papillose, which is a unique feature within the genus. The nrDNA sequences did not provide heterogeneity that excluded recent hybrid origins and possibly increased the size of the plants; however, the robust support for evidence of autopolyploidization could not be excluded or confirmed because a special karyological study could not be performed within the present research.

The long (with a significantly longer length than widths) cells of the leaf segments, with perpendicularly protruding cell walls, make this species vaguely morphologically similar to *B. neglectum*, which, at the southernmost of its known range, reaches Yunnan and Sichuan Provinces, China [2]. This morphological similarity is supported by the sister relationships in the phylogenetic trees (Figure 1 and Figure 2). In addition to the general differentiating characteristics mentioned above, *B. neglectum* never develops small homogeneous oil bodies, and large oil bodies are of grape-cluster type but not finely papillose.

Additionally, it is necessary to note the size of the plants. In tropical mountain climates, the rate of plant growth should increase compared with that in the northern Holarctic, where *Blepharostoma* are quite abundant, and in this case, the new species may have an advantage over other species of the genus due to the larger size of the plants, which allows it to be more successful in competing with other liverworts inhabiting wet rocks, a very popular habitat for liverworts in the mountains near the Tropic of Cancer as well as in the North Holarctic.

### 3.2. Ecology

All the specimens were collected at the upper part of the altitudinal gradient of the Hoang Lien Son Range (from 2591 to 2846 m a.s.l.). This area, following Averyanov et al. [4], is located in the zone of dominance of the monsoon tropical climate associated with the mountains, some parts of which are characterized by high precipitation amounts exceeding 3500 mm per year [4]. However, with respect to the bioclimatic indicators measured along the transect passing through the summit of Phan Xi Pan Mountain, the highest point of the range, the maximum precipitation reaches only 2162 mm per year and decreases toward the ridge line, where it is only 1780 mm per year [5]. Thin and Harder [6] refer to the vegetation at altitudes above 2000 m a.s.l. in the area to the temperate vegetation belt that is represented by temperate forest and montane cold savanna. This statement seems questionable to us, but there is no doubt about the presence of many predominantly temperate genera in this altitude range, which is also confirmed by the list of species provided in the cited source. The same point of view regarding the temperate vegetation belt is reflected in Nguyen and Nguyen [7]. In support of their point of view, the authors list the genera that include predominantly temperate genera occurring in Hoang Lien: *Abies*, *Acer*, *Adinandra*, *Aesculus*, *Agapetes*, *Alnus*, *Altingia*, *Coptis*, *Cornus*, *Crawfurdia*, *Embelia*, *Enkianthus*, *Fagus*, *Fokienia*, *Hydrangea*, *Huodendron*, *Rehderodendron*, *Liriodendron*, *Magnolia*, *Oxyspora*, *Primula*, *Quercus*, *Rhododendron*, *Rhoiptelea*, *Sorbus*, *Ternstroemia*, *Vaccinium,* and *Valeriana*.

All the specimens we have at our disposal were collected in scattered *Rhododendron* forests (with the participation of many mountain subtropical plants) with a dense understory of *Sinobambusa sat* (Balansa) C.S. Chao & Renvoize. In one instance, the habitat also included scattered *Abies fansipanensis* Q.P. Xiang, L.K. Fu, & N. Li (=*Abies delavayi* subsp. *fansipanensis* (Q.P. Xiang, L.K. Fu, & N. Li) Rushforth), an endemic species in the Hoang Lien Son Range. *Blepharostoma vietnamicum* grows on partially shaded to open, moderately moist granite rocks over mosses (including *Sphagnum* sp.) and liverworts. The same growing conditions are common for almost all other *Blepharostoma* species. Among liverworts, the new species was associated with *Bazzania himalayana* (Mitt.) Schiffn., *B. ovistipula* (Steph.) Abeyw., *Fuscocephaloziopsis gollanii* (Steph.) Váňa & L. Söderstr., *Heteroscyphus tener* (Steph.) Schiffn., *Radula cavifolia* Hampe ex Gottsche, Lindenb. & Nees, *Scapania ornithopoides* (With.) Waddell, *Schistochilopsis setosa* (Mitt.) Konstant. and presumably new-to-science, *Riccardia* sp. If the species names of the listed genera are set aside, the same genera are characteristic companions of *Blepharostoma* species that grow on wet rocks in the northern parts of the Holarctic; therefore, the basic genus ecology is not changed. At the same time, the vast majority of the listed taxa, if not limited, have an area core in the Sino-Himalayas, and their distribution on the Hoang Lien Son Range is located on the southern flanks of their ranges. All these species can be classified as oro-temperate taxa; B. *vietnamicum* also most likely belongs to that group. On the basis of its ecological requirements, this species can be considered an acidophilic mesophyte, similar to most other *Blepharostoma*.

### 3.3. Possible Geographic Distribution

*Blepharostoma* rarely penetrates south of the Sino-Himalayas in Asia. Although *B. trichophyllum* s.l. is known from Malaya, Borneo, the Philippines, Java, and New Guinea [8,9,10,11], this “taxon” is rare there. In addition, it is generally not clear which species are found in Malesia and Melanesia. The plants that were observed in Malesia and Melanesia most likely belong to the taxa that are not yet described but not to the “true” *B. trichophyllum* s. str. Until now, only one species of *Blepharostoma* was known in Indochina, *B. minus*. Moreover, the species identity of the sampled *B. minus* plants was proven via molecular genetic studies [2]. Interestingly, this species is known in approximately the same area from which *B. vietnamicum* was collected.

*Blepharostoma vietnamicum* was collected from the Hoang Lien Son Range, which is generally known as a landmark site for the southward penetration of more northernly distributed Sino-Himalayan species [4,12,13]. At the same time, this range is also a place where probable endemic taxa are distributed, which are hitherto unknown in other regions. Thus, both a narrow distribution of the taxon and a wider, at least Eastern Sino-Himalayan, distribution seem equally likely. Given that the species has been collected several times, it should not be rare in the Hoang Lien Son Range, meaning that future research will likely identify it in other areas of northern Vietnam.

In the upper belt of the Hoang Lien Son Range (i.e., above 2000 m above sea level), the following liverworts have been described and are still known only to be present within that range: *Calypogeia vietnamica* Bakalin & Vilnet; *Gymnomitrion vietnamicum* Bakalin & Vilnet; *Marsupella anastrophylloides* Bakalin, Vilnet & Maltseva; *M. vietnamica* Bakalin & Fedosov; and *Vietnamiella epiphytica* Bakalin & Vilnet. In addition, from the lower levels of the Hoang Lien Son Range, only *Frullania pocsantha* Thaithong & S. Hatt. is described and restricted to it, as far as we know. Thus, the liverwort flora of the upper altitude level is much more specific than the one of the lower altitude levels. However, for vascular plants, the situation is exactly the opposite, as follows from the calculation by Vu and Nguyen [14]. There are 122 endemic species (comprising 5% of the total number of vascular species) belonging to 52 vascular plant families in the Hoang Lien Son Range. Most of these species (96 species or 76%) are distributed in the altitude range of 1000–1700 m a.s.l. Thus, more endemics are known from the lower belt than from the upper belt. *Blepharostoma vietnamicum* likely belongs to the group as the suggested upper-level endemic taxa.

## 4. Materials and Methods

### 4.1. Herbarium Materials

The material for this work was collected during joint Russian–Vietnamese North Vietnam liverwort diversity exploration in 2018 and 2023. When the specimens were collected, the geographic coordinates, altitude above sea level, type of vegetation community, and ecological habitat conditions, including moisture supply and illumination, were recorded. In 2018, the collected specimens were delivered live to the Laboratory of Cryptogamic Biota of the Botanical Garden Institute of the Far Eastern Branch of the Russian Academy of Sciences (Vladivostok), where they were photographed using stationary digital cameras mounted on Olympus CX31 and SZX16 microscopes. The specimens collected during the 2023 expedition were studied at the Laboratory of Plant Ecology at the Institute of Ecology and Biological Resources of the Vietnam Academy of Science and Technology (Hanoi), and intravital photographs were taken using digital cameras mounted on Nikon SMZ800N and Olympus BX43 microscopes in the latter laboratory. During the initial study of living material, several characteristics were noted and photographed (e.g., plant size and morphology of the oil bodies), and some plants were isolated in dry silica gel-filled plastic zip-lock bags for molecular genetic research. A detailed morphological description was subsequently compiled after molecular genetic analysis was performed. At the same time, a morphological and anatomical comparison of the plants in the studied specimens with species already known within the genus was made, information regarding the ecology was examined, and the potential distribution of the new taxon was assessed.

All the specimens were collected from the upper elevations at the southern tip of the Hoang Lien Son Range, which is a very distinctive phytogeographical area where Sino-Himalayan floral elements penetrate Indochina [12] and where a large group of endemic species are concentrated [4]. The collection areas are located in Hoang Lien National Park, in the Phan Xi Pan Mountain—the so-called “Roof of Indochina”—the highest point of all Indochina. The history of liverwort studies in this area, its phytogeographical significance for understanding the characteristics of the flora of Indochina, and the list of liverwort species known in the park and near its environs were provided previously [13].

### 4.2. Materials for Molecular Analysis

For the molecular phylogenetic estimation of unique plants, two specimens from different localities were selected: V-61-28-23 and V-66-27a-23. The appropriate dataset included specimens of elderly known and recently described species of the genus *Blepharostoma* that were sampled from the closest Asian territories: *B. brevirete* (Bryhn & Kaal.) Vilnet & Bakalin; *B. primum* Vilnet & Bakalin; *B. pseudominus* Vilnet & Bakalin; *B. trichophyllum* and *B. trichophyllum* hybrid taxon 1 from the Russian Far East; *B. neglectum* Vilnet & Bakalin from the Russian Far East and China; *B. epilithicum* Vilnet & Bakalin from Japan and South Korea; and *B. minus* from the Russian Far East, South Korea, Japan, and Vietnam. Additionally, *B. arachnoideum* M. Howe, with a North American distribution, as well as undescribed possibly cryptic taxa that were collected only once from the Khabarovsk Territory of Russia, were added to the estimation. In total, 29 specimens of *Blepharostoma* from Bakalin et al. [2] were placed on the basis of molecular genetic research. The four taxa from phylogenetically allied families [15] are chosen as additional members of the ingroup to highlight the generic framework of *Blepharostoma*: *Herbertus delavayi* (Steph.) Steph. (Herbertaceae); *Plagiochila xerophila* Bakalin & Vilnet (Plagiochilaceae); *Ceramanus clatritexta* (Steph.) E.D. Cooper (Lepidoziaceae); and *Vetaforma dusenii* (Steph.) Fulford & J. Taylor (Lepicoleaceae). *Marsupella taiwanica* Mamontov, Vilnet & Schäf.-Verw. (Gymnomitriaceae) from another order (Jungermanniales) was used as an outgroup taxon. Two genomic loci, ITS1-2 nrDNA and *trn*L-F cpDNA, were selected as molecular markers in a previous study of the genus [2]. The list of 36 specimens that were included in the phylogenetic estimation with voucher details and GenBank accession numbers is shown in Table 1.

### 4.3. DNA Isolation, Amplification and Sequencing

The DNA was extracted from the liverwort shoots that were dried in silica gel with a DNeasy Plant Mini Kit (QIAGEN) according to the manufacturer’s protocol. For the amplification and sequencing procedures, pairs of primers were used, as suggested by White et al. [16] for ITS1-2 and Taberlet et al. [17] for *trn*L-F. PCR was carried out in 20 μL volumes with the following procedure: 3 min at 94 °C, 30 cycles (30 s at 94 °C, 40 s at 56 °C, and 60 s at 72 °C), and 2 min with a final extension at 72 °C. The obtained fragments were visualized on 1% agarose TAE gels via EthBr staining. The amplicons were cleaned from agarose with a Cleanup Mini Kit (Evrogen, Moscow, Russia) and then sequenced with the ABI Prism BigDye Terminator Cycle Sequencing Ready Reaction Kit (Applied Biosystems, Waltham, MA, USA) following the standard protocol provided for the 3730 DNA Analyzer (Applied Biosystems, Waltham, MA, USA) at the Genome Center of EIMB (Moscow, Russia).

### 4.4. Phylogenetic Analysis

Sequence assembly and dataset alignment were performed in BioEdit 7.0.1 [18] with the automatic ClustalW tool and subsequent manual corrections. At least two hybridization events were detected in the genus *Blepharostoma,* with the origin of *B. neglectum* and *B. trichophyllum* hybrid taxon 1 from two still unknown possibly extinct taxa and *B. trichophyllum*, which became a maternal parent in the first case and a paternal parent in the second case [2]. This prevents us from combining the ITS1-2 and *trn*L-F datasets into one dataset to avoid conflicting phylogenetic signals. Phylogenetic calculations were provided for both datasets separately, with all positions included in the analyses. Maximum likelihood (ML) analyses with IQTree [19] and the Bayesian approach with MrBayes v. 3.2.1 [20] were implemented to test the phylogeny of the genus *Blepharostoma*. The search for the best-fitting evolutionary model for the nucleotide substitutions was performed in ModelFinder [21]. The TIM3 + F + I + G4 model was chosen for the ITS1-2 dataset, and the HKY + F + G4 model was chosen for the *trn*L-F dataset. The ultrafast bootstrapping procedure [22] revealed that 123 iterations were sufficient to search for the best tree from the ITS1-2 dataset, and 149 iterations were sufficient for the *trn*L-F dataset. The ML tree topologies were visualized in NJplot [23]. The Bayesian calculations for both datasets included the GTR + I + G model, as recommended by the program manufacturers, with gamma distributions with four rate categories. Chains of two independent runs of the metropolis-coupled ΜCMC were run for one million generations for the ITS1-2 and *trn*L-F datasets. Trees were sampled every 100th generation. The first 2500 trees in each run were discarded as burn-in, and 15,000 trees were sampled from both runs for each dataset. The average standard deviation of the split frequencies between two runs for the ITS1-2 dataset was 0.004193; for *trn*L-F, it was 0.004862. Bayesian posterior probabilities were calculated from the trees that were sampled after burn-in. The BA tree topologies were redrawn in FigTree v1.3.1 [24]. The levels of interspecific ITS1-2 and *trn*L-F nucleotide sequence divergences and intraspecific variability in the genus *Blepharostoma* were estimated as average pairwise *p*-distances using the pairwise deletion option for counting gaps in Mega 11 [25].

## 5. Conclusions

New-for-science taxa often provide the researcher with unexpected combinations of features. Among *Blepharostoma*, a previously known species, neither large finely granulate oil bodies nor their combination with small homogeneous oil bodies within a single cell were observed. Moreover, the described species combines both types of oil bodies within a single cell. Additionally, it was not previously assumed that representatives of the genus *Blepharostoma* can reach 1.5 mm in width, which makes it similar to other genera from other families. Thus, the present new species is not only new in the combination of features previously unknown within the genus but also represents new features previously unknown within the genus. The latter makes promising further investigation of *Blepharostoma* morphology.

Among the vascular plants, the largest number of endemics in the Hoang Lien Son Range is concentrated in the lower vegetation zone, while the narrowly distributed liverworts are concentrated in the upper altitudes. A logical question arises as to the extent to which those species that we classify as endemic to Hoang Lien Son Range are actually endemic and whether they are not all actually Sino-Himalayan taxa that were just described from the mountains of northern Vietnam. The knowledge of the distribution of liverworts is much more fragmented than that of vascular plants. However, the observed pattern is a strong reason to wonder whether this finding indicates more serious pattern differences from those observed in vascular plants. There is no doubt that further liverwort research in the Hoang Lien Son Range will reveal new species for science and inspire a more complete understanding of the Hoang Lien liverwort phenomenon.

## Figures and Tables

**Table 1:** The list of specimens included in the current phylogenetic estimation, including voucher details and GenBank accession numbers. The sequences obtained in this study are shown in bold.

Taxon	Specimen Voucher	GenBank Accession Number	
		ITS1-2 nrDNA	*trn*L-F cpDNA
*Blepharostoma arachnoideum* M. Howe	USA: Oregon, D. Wagner, SM-2019-25 (VBGI)	MT586201	MT585796
*B. arachnoideum*	USA: Oregon, D. Wagner, GV18-1047 (VBGI)	MT586205	MT585799
*Blepharostoma brevirete* (Bryhn & Kaal.) Vilnet & Bakalin	Russia: Kamchatka Prov., V. Bakalin, K-50-11-15, 300157 (VBGI), 123100 (KPABG)	MT586189	MT585782
*B. brevirete*	Russia: Magadan Prov., V. Bakalin, Mag-10-1-10, 313792 (VBGI), 115163 (KPABG)	MT586190	MT585783
*Blepharostoma epilithicum* Vilnet & Bakalin	Japan: Kochi, V. Bakalin, J-11-13-15 (VBGI), 123110 (KPABG)	MT586185	MT585777
*B. epilithicum*	South Korea: Gangwon Prov., S.-S. Choi, 37-10, 115503 (KPABG)	MT586186	MT585778
*Blepharostoma minus* Horik.	Japan: Miyazaki Pref., V. Bakalin, J-91-51-15, 303782 (VBGI), 123087 (KPABG)	MT586179	MT585770
*B. minus*	Russia: Sakhalin Prov., Kuril I., Shikotan Isl., V. Bakalin, K-49-6-07, 313369 (VBGI), 115152 (KPABG)	MT586181/ MT586210	MT585772
*B. minus*	South Korea: Gyeongnam Prov., V Bakalin, Kor-16-19a-09, 317099 (VBGI), 115153 (KPABG)	MT586183	MT585775
*B. minus*	Vietnam: Lao Cai Prov., V. Bakalin, K. Klimova, V-1-27-17, 35095 (VBGI), 122638 (KPABG)	MT586184	MT585776
*Blepharostoma neglectum* Vilnet & Bakalin	China: Sichuan Prov., V. Bakalin, K. Klimova, China-44-10-17, 37380 (VBGI), 122632 (KPABG)	MT586150	MT585736
*B. neglectum*	China: Yunnan Prov., V. Bakalin, C-77-18-18 (VBGI), 123112 (KPABG)	MT586151	MT585737
*B. neglectum*	Russia: Khabarovsk Terr., V. Bakalin, Kh-46-3-19 (VBGI), 123108 (KPABG)	MT586153	MT585738
*B. neglectum*	Russia: Magadan Prov., V. Bakalin, Mag-18-4-10, 313978 (VBGI), 115164 (KPABG)	MT586155	MT585740
*B. neglectum*	Russia: Primorsky Terr., V. Bakalin, P-56-10-08, 115148 (KPABG)	MT586159	MT585743
*B. neglectum*	Russia: Sakhalin Prov., Kuril I., Iturup Isl., V. Bakalin, K-32-15-07, 311588 (VBGI), 115160 (KPABG)	MT586165	MT585749
*Blepharostoma primum* Vilnet & Bakalin	Russia: Khabarovsk Terr., V. Bakalin, Kh-47-28-19 (VBGI), 123111 (KPABG)	MT586195	MT585790
*B. primum*	Russia: Primorsky Terr., E. Borovichev, G116245 (KPABG)	MT586197	MT585792
*B. primum*	Russia: Sakhalin Prov., V. Bakalin, S-49-13-09, 309815 (VBGI), 115158 (KPABG)	MT586200	MT585795
*Blepharostoma pseudominus* Vilnet & Bakalin	Russia: Primorsky Terr., V. Bakalin, P-4-1-08, 310194 (VBGI), 115159 (KPABG)	MT586192	MT585785
*B. pseudominus*	Russia: Sakhalin Prov., Kuril I., Kunashir Isl., V. Bakalin, K. Klimova, K-44-21-18, 57923 (VBGI), 122499 (KPABG)	MT586194	MT585789
*Blepharostoma* sp. (cryptic taxon)	Russia: Khabarovsk Terr., V. Bakalin, Kh-40-31-13, 302859 (VBGI), 123104 (KPABG)	MT586178	MT585769
*Blepharostoma trichophyllum* (L.) Dumort.	Russia: Kamchatka Terr., V. Bakalin, K-66-13-15, 300362 (VBGI), 123090 (KPABG)	MT586139	MT585727
*B. trichophyllum*	Russia: Khabarovsk Terr., V. Bakalin, Kh-50-17-19 (VBGI), 123107 (KPABG)	MT586142	MT585730
*B. trichophyllum*	Russia: Magadan Prov., V. Bakalin, Mag-29-26-14, 301897 (VBGI), 123088 (KPABG)	MT586135	MT585723
*B. trichophyllum*	Russia: Sakhalin Prov., V. Bakalin, S-30-9-09, 309613 (VBGI), 115162 (KPABG)	MT586144	MT585732
*Blepharostoma trichophyllum* hybrid taxon 1 sensu Bakalin et al. (2020)	Russia: Khabarovsk Terr., V. Bakalin, Kh-58-4-19 (VBGI), 123106 (KPABG)	MT586171	MT585762
*B. trichophyllum* hybrid taxon 1	Russia: Magadan Prov., V. Bakalin, Mag-57-2-11, 316644 (VBGI), 123094 (KPABG)	MT586174	MT585765
*B. trichophyllum* hybrid taxon 1	Russia: Sakhalin Prov., V. Bakalin, S-32-27a-06, 115166 (KPABG)	MT586176	MT585767
*Blepharostoma vietnamicum* Bakalin, Vilnet et S.S. Choi sp.nov.	Vietnam: Lao Cai Prov., V. Bakalin, V-61-28-23, 205851 (VBGI)	PP479835	PP480520
*B. vietnamicum*	Vietnam: Lao Cai Prov., V. Bakalin, V-66-27a-23, 206059 (VBGI)	PP479836	PP480521
*Ceramanus clatritexta* (Steph.) E.D. Cooper	Australia, E.D. Cooper 09/052	JX289145	JX289427
*Herbertus delavayi (*Steph.) Steph.	Bhutan, D. Long, 28864 (H)	KU523787	KU523722
*Marsupella taiwanica* Mamontov, Vilnet & Schäf.-Verw.	Taiwan: Chiayi Co., A. Schafer-Verwimp, 39136 (MHA), 123645 (KPABG)	OM509628	OM515127
*Plagiochila xerophila* Bakalin & Vilnet	China: Sichuan Prov., V. Bakalin, K. Klimova, China-46-2-17, 37198 (VBGI), 122589 (KPABG)	MK121889	MK123266
*Vetaforma dusenii* (Steph.) Fulford & J. Taylor	Chile, J.J. Engel, 11423 (H)	DQ293965	AY463593

**Table 2:** The infra- and interspecific *p*-distances for the species of the genus *Blepharostoma* were obtained from ITS1-2 and *trn*L-F.

Taxon	Infraspecific *p*-Distances, ITS1-2/*trn*L-F, %	Interspecific *p*-Distances, ITS1-2/*trn*L-F, %										
		*viet.*	*neg.*	hyb. 1	*trich.*	sp.	*min.*	*epil.*	*brev.*	*pseud.*	*prim.*	*arch.*
*B. vietnamicum*	0.0/0.0											
*B. neglectum*	2.1/0.6	6.8/2.8										
*B. trichophyllum* hybrid taxon 1	0.6/0.3	9.2/2.5	7.9/2.8									
*B. trichophyllum*	0.5/0.4	9.0/2.6	7.8/0.5	0.9/2.6								
*Blepharostoma* sp.	n/c/n/c	7.6/3.3	6.8/3.5	6.7/2.6	6.5/3.4							
*B. minus*	1.1/0.1	10.9/4.0	10.5/4.1	10.5/3.1	10.4/3.9	9.5/3.6						
*B. epilithicum*	0.6/1.0	8.1/3.9	7.4/3.7	7.6/3.2	7.3/3.4	6.8/3.6	9.3/3.3					
*B. brevirete*	0.0/0.2	7.7/2.4	6.7/2.6	6.9/1.7	6.7/2.4	5.8/2.0	8.6/2.6	6.0/2.6				
*B. pseudominus*	0.2/0.4	10.9/4.1	9.5/4.4	10.1/3.5	9.8/4.2	9.5/3.9	11.5/3.9	10.3/4.5	8.2/3.0			
*B. primum*	0.2/0.4	16.6/8.7	15.9/9.3	16.0/8.4	15.8/9.0	16.6/8.0	18.0/9.0	15.9/8.8	14.9/8.1	16.3/9.7		
*B. arachnoideum*	0.0/0.4	20.1/7.4	19.0/8.5	19.2/7.6	19.2/8.3	20.0/7.4	20.6/8.8	20.1/8.9	19.0/7.2	18.7/9.1	21.3/8.7	

## Data Availability

All data are contained within the article.
